# Clinical versus Sonographic Estimation of Foetal Weight in Southwest Nigeria

**Published:** 2007-03

**Authors:** Akinola S. Shittu, Oluwafemi Kuti, Ernest O. Orji, Niyi O. Makinde, Solomon O. Ogunniyi, Oluwagbemiga O. Ayoola, Salami S. Sule

**Affiliations:** ^1^ Department of Obstetrics and Gynaecology; ^2^ Department of Radiology; ^3^ Department of Community Health, Obafemi Awolowo University Teaching Hospital Complex, Ile-Ife, Osun State, Nigeria

**Keywords:** Foetal weight, Birth-weight, Ultrasonography, Pregnancy, Delivery, Prospective studies, Comparative studies, Nigeria

## Abstract

A prospective study was conducted at Obafemi Awolowo University Teaching Hospital Complex, Ile-Ife, Nigeria, between 3 January and 31May 2004, to compare the accuracy of clinical and ultrasonographic estimation of foetal weight at term. One hundred pregnant women who fulfilled the inclusion criteria had their foetal weight estimated independently using clinical and ultrasonographic methods. Accuracy was determined by percentage error, absolute percentage error, and proportion of estimates within 10% of actual birthweight (birthweight of +10%). Statistical analysis was done using the paired *t*-test, the Wilcoxon signed-rank test, and the chi-square test. The study sample had an actual average birthweight of 3,255+622 (range 2,150–4,950) g. Overall, the clinical method overestimated birthweight, while ultrasound underestimated it. The mean absolute percentage error of the clinical method was smaller than that of the sonographic method, and the number of estimates within 10% of actual birthweight for the clinical method (70%) was greater than for the sonographic method (68%); the difference was not statistically significant. In the low birthweight (<2,500 g) group, the mean errors of sonographic estimates were significantly smaller, and significantly more sonographic estimates (66.7%) were within 10% of actual birthweight than those of the clinical method (41.7%). No statistically significant difference was observed in all the measures of accuracy for the normal birthweight range of 2,500-<4,000 g and in the macrosonic group (≥4,000 g), except that, while the ultrasonographic method underestimated birthweight, the clinical method overestimated it. Clinical estimation of birthweight is as accurate as routine ultrasonographic estimation, except in low-birthweight babies. Therefore, when the clinical method suggests weight smaller than 2,500 g, subsequent sonographic estimation is recommended to yield a better prediction and to further evaluate foetal well-being.

## INTRODUCTION

Accurate estimation of foetal weight is of paramount importance in the management of labour and delivery. During the last decade, estimated foetal weight has been incorporated into the standard routine antepartum evaluation of high-risk pregnancies and deliveries. For instance, management of diabetic pregnancy, vaginal birth after a previous caesarean section, and intrapartum management of foetuses presenting by the breech will be greatly influenced by estimated foetal weight (1, 2).

Also, when dealing with anticipated preterm delivery, perinatal counselling on likelihood of survival, the intervention undertaken to postpone preterm delivery, optimal route of delivery, or the level of hospital where delivery should occur may be based wholly or in part on the estimation of expected birthweight. Categorization of foetal weight into either small or large for gestational age may lead to timed obstetric interventions that collectively represent significant departure from routine antenatal care (2, 3–5). High rate of perinatal mortality (39–130 per 1,000 total births) is still a major cause for concern in developing countries such as Nigeria (8). A large portion of this problem is related to birthweight which remains the single most important parameter that determines neonatal survival (6–9).

It is estimated that 16% of liveborn infants have low birthweight, a condition associated with high perinatal morbidity and mortality. Foetal macrosomia is associated with maternal morbidity, shoulder dystocia, birth asphyxia, and birth trauma (10). An incidence of 1.6% of macrosomia was quoted in Obafemi Awolowo University Teaching Hospital Complex, Ile-Ife, in 1991, while 4.9% was reported in 1983 to 1985 series in the Lagos University Teaching Hospital (11).

It has been suggested that accurate estimation of foetal weight would help in successful management of labour and care of the newborn in the neonatal period and help avoidance of complications associated with foetal macrosomia in low-birthweight babies, thereby decreasing perinatal morbidity and mortality (2–4, 12–21).

The two main methods for predicting birthweight in current obstetrics are: (a) clinical techniques based on abdominal palpation of foetal parts and calculations based on fundal height and (b) sonographic measures of skeletal foetal parts which are then inserted into regression equations to derive estimated foetal weight (4–6). Although some investigators consider sonographic estimates to be superior to clinical estimates, others, in comparing both the techniques concurrently, conclude that they confer similar levels of accuracy (3–7, 12–46).

The available techniques can be broadly classified as: (a) clinical methods: tactile assessment of foetal size, e.g. Leopold's manoeuvre; clinical risk factor; maternal self-estimated foetal weight; and prediction of equations of birthweight and (b) imaging methods: ultrasonography and magnetic resonance imaging.

**Tactile assessment of foetal size:** Dare *et al.* used this technique (21). It is the oldest technique for assessing foetal weight through manual assessment of foetal size by obstetricians worldwide, i.e. by external palpation of the uterus and foetal parts. This method is extensively used because it is both convenient and virtually costless. However, it has long been known as a subjective method that is associated with significant predictive errors. It is both patient- and clinician-dependent for its success (less accurate for obese gravidas than non-obese and significant inter-observer variation in prediction of birthweight even among experienced clinicians) (27).

**Clinical risk factor:** This involves quantitative assessment of clinical risk factors and has been shown to be valuable in predicting foetal weight. In the case of foetal macrosomia, the presence of risk factors, such as maternal diabetes mellitus, abnormal glucose screening test, prolonged pregnancy, maternal obesity, pregnancy-weight gain of >20 kg, maternal age of >35 years, maternal height >5 ft 3 in, multiparity, male foetal sex, and white race, should make the obstetrician suspicious of foetal macrosomia and assess accordingly.

**Maternal self-estimation:** Perhaps surprisingly in developed (literate) society, maternal self-estimation of foetal weight in multiparous women shows comparable accuracy to clinical palpation in some studies for predicting abnormally large foetuses (24, 29).

**Birth-weight prediction equations:** Various calculations and formulae based on measuring uterine fundal height above symphysis pubis have been developed. Ojwang *et al.* used the product of symphysiofundal height and abdominal girth measurement at various levels in centimetres above the symphysis pubis in obtaining a fairly acceptable predictive value but with considerable variation from the mean (20). To further simplify this method, Dare *et al.* in OAUTHC, Ile-Ife, in 1988, used the product of symphysiofundal height and abdominal girth at the level of the umbilicus measured in centimetres and result expressed in grammes to estimate foetal weight at term in-utero, and the estimate correlated well with birthweight (21).

Johnson's formula for estimation of foetal weight in vertex presentation is as follows: Foetal weight (g)=fH (cm)n × 155. fH=fundal height and n=12 if vertex is above ischial spine or 11 if vertex is below ischial spine. If a patient weighs more than 91 kg, 1 cm is subtracted from the fundal height.

**Predicting foetal weight using algorithm derived from maternal and pregnancy-specific characteristics.** Recently, a new theoretically-defensible equation that can predict individual birthweight prospectively from maternal characteristics was developed. To do this, the efficacy of 59 scientifically-justifiable terms was evaluated simultaneously, obviating any confounding co-variation and determining which of the predictions could account for variation in birthweight that others could not. Aside from maternal race, only six maternal and pregnancy-specific variables were important in prediction of birthweight for otherwise normal gravidas. Using these routinely-recorded variables, an equation, based on maternal demographic and pregnancy-related characteristics alone, was developed to help predict birthweight as follows:

Birth-weight (g)=gestational age (d) × [9.36 + 0.262 × foetal sex + 0.000237 × maternal height (cm) × maternal weight at 26 weeks (kg) + (4.81 × maternal weight gain rate (kg/d) × (parity+1)], where foetal sex is equal to +1 for male, -1 for female, and 0 for unknown sex, and gestational age is equal to days since onset of last normal menses which equals the conception age (d)+14 (10).

**Obstetric ultrasonography.** A modern method for assessing foetal weight involves the use of foetal measurement obtained via ultrasonography. The advantage of this technique is that it relies on linear and/or planar measurement of in-utero foetal dimensions that are definable objectively and should be reproducible. Early expectation that this method might provide an objective standard for identifying foetuses of abnormal size for gestational age was recently undermined by prospective studies that showed sonographic estimates of foetal weight to be no better than clinical palpation for predicting foetal weight (26, 27, 34).

Susuki *et al.* used ultrasound measurement of foetal heart volume to estimate foetal weight (44), while Paulos *et al.* used foetal volume by ultrasound (45). Today, sonographic predictions are based on algorithms using various combinations of foetal parameters, such as abdominal circumference (AC), Femur length (FL), biparital diameter (BPD), and head circumference (HC) both singly and in combination as shown below (3, 10, 18, 25, 37–42)

**Table:** 

Source	Year	Equation
Shepard	1983	Log_10_BW=1.7492+0.0166(BPD^+^) + 0.0046(AC) - 0.00002646 (ACxBPD)
Campbell	1975	LnBW=4.564+0.0282 (AC)-0.0000331(AC)^2^
Hadlock I	1985	Log_10_BW=1.326–0.0000326 (ACxFL) × 0.00107(HC) + 0.00438 (AC) + 0.0158(FL)
Hadlock 2	1985	Log_10_BW=1.304+0.005251(AC) + 0.01938 (FL) 0.00004(Acx FL)
Hadlock 3	1985	Log_10_BW=1.335–0.000034(ACxFL)+0.00316x (BPD)+0.0045 (AC)+0.01623 (FL)
Warsof 1	1986	LnBW=4.6914+0.00151(FL)^2^- 0.0000119 (FL)^3^
Warsof 2	1986	LnBW=2.792+0.108 (FL)+0.000036 (AC)^2^-0.00027 (FLXAC)
Combs	1993	BW=(0.00023718x(AC)^2^x(FL)^2^)+0.00003312(HC)^3^
Ott	1986	Log_10_BW=0.004355(HC)+0.005394 (AC)-0.00008582 (HCx AC)+1.2594 (FL/AC)-2.0661
Nzeh *et al.* (formula1)	1992	Log_10_BW=0.470+0.488 Log_10_BPD+0.554 Log_10_ FL+1.377 Log_10_AC
Nzeh *et al.* (formula 2)	1992	Log_10_ BW=0.326+0.00451(SDI)+0.383 Log_10_BPD+0.614 Log_10_FL+1.485Log_10_AC
Deter	1985	EFW=10^1.335–0.0034AcxFL+0.0316BPD +0.0457AC+0.1623FL^

Obstetric sonographic assessment for the purpose of obtaining foetal biometric measurement to predict foetal weight has been integrated into the main stream of obstetric practice during the past quarter century. The above modern algorithms are generally comparable in terms of overall accuracy in predicting birthweight. When other sonographic foetal measurements are used for estimating foetal weight, e.g. humeral soft tissue thickness, ratio of subcutaneous tissue to femoral length, cheek-to-cheek distance, these non-standard measurements do not significantly improve the ability of obstetric sonography to help predict birthweight, except in special patients subgroup, e.g. mothers with diabetes (32).

The notion that multiple obstetric sonographic foetal biometric evaluation might prove superior to a single examination has also been assessed and has not been found to be helpful (25, 26).

Several technical limitations of the sonographic technique for estimating foetal weight are well-known. Among these are maternal obesity, oligohydramnios, and anterior placentation. Other disadvantages of ultrasonography are that it is both complicated and labour intensive, potentially being limited by suboptimal visualization of foetal structure. It also requires costly sonographic equipment and specially trained personnel. Although such expensive imaging equipment is widely available in developed countries, this is generally not the case in developing nations like ours where medical resources are scarce (12, 25).

**Magnetic resonance imaging:** This has recently been used for estimating foetal volume and weight in diabetic and normal pregnancy using high-resolution magnetic resonance imaging machine combined with a semi-automatic segmentation software. Its use may be recommended for clinical situation where accurate estimation is essential. Its strong disadvantage is that even where it is available it is expensive (28).

All currently-available techniques for estimating foetal weight have significant degree of inaccuracy, and various studies have been done to compare the accuracy of different methods of estimation. Limiting the potential complications associated with birth of both small and excessively large foetuses requires that accurate estimation of foetal weight occurs in advance of deliveries (3–5).

This study aims at resolving these controversies and at determining the more accurate method of foetal-weight estimation of the two in our environment, thereby improving management of conditions earlier mentioned.

## MATERIALS AND METHODS

### Study population

This prospective comparative study was carried out at the Obstetrics and Gynaecology Department of Obafemi Awolowo University Teaching Hospital Complex, Ile-Ife, Osun State, Nigeria, between 3 January and 31 May 2004.

The study subjects were mothers with singleton pregnancy admitted for planned delivery at term for various reasons either by elective caesarean section or by induction of labour. One hundred consecutive pregnant women who fulfilled the inclusion criteria were counselled and, after consenting, were included in the study. The women had their gestational age confirmed by dates and ultrasound scanning before 22 weeks and were managed according to laid down departmental protocols.

The exclusion criteria were obese patients (weight more than 90 kg), patients with polyhydramnios, pre-term labour, ruptured membranes, abnormal lie and presentation, multiple pregnancies, antepartum haemorrhage, eclampsia, obvious congenital abnormalities, oligohydramnios, anteriorly-inserted placenta, and poor visualization of foetal part.

The interval between clinical and ultrasound estimation of foetal weight in-utero and delivery of babies was within 24 hours. Only the senior resident assigned to the labour ward carried out in-utero estimation of foetal weight using the same flexible tape measure calibrated in centimetres. Using this tape, fundal height was measured from the highest point on the uterine fundus to the midpoint of the upper border of the symphysis pubis, using the thumb to sustain the tape, while attempting to reach the upper border of the symphysis pubis, measurement was made using the tape reverse-side up so as to forestall any bias. The abdominal circumference was also measured at the umbilicus level. Fundal height multiplied by abdominal girth measurement in centimetres was used for calculating foetal weight in grammes. The patient was then sent for ultrasonographic estimation done by a senior resident of the radiology unit using an abdominal sector 3.5 MHz transducer on the Sonace 3200 ultrasound machine designed by Advanced Technology Laboratories, Bothell, WA, Australia. Its formula for estimating foetal weight is that devised by Hadlock (3) on the basis of biparietal diameter (BPD), abdominal circumference (AC), and femural length (FL) (18). The sonologist had no prior knowledge of the clinical estimate of foetal weight. Both the estimates were documented into a chart. After delivery, experienced midwives weighed newborn babies within 30 minutes of delivery employing a standard analogue Waymaster (England) scale corrected for zero error.

### Determination of sample size

The sample size was determined using the Computer Programme for Epidemiologist (PEPI), version 3.01, described by Armitage and Berry, and cited in Gahlinger and Abramson (1999) (33) employing the formula:

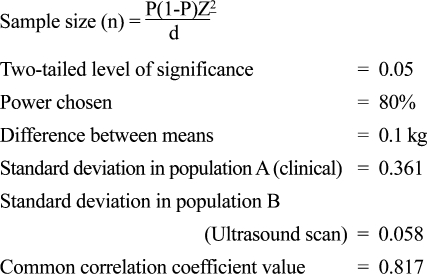


The sample size obtained was 75. However, 100 consecutive patients were considered for increasing the power of the study. All data obtained during the study period were entered into a collection form specifically designed for the study.

### Statistical analysis

Accuracy of birthweight was determined by calculating the percentage error (EFW-ABW) × 100/ABW, the absolute error, i.e. [absolute value (EFW-ABW)] × 100/ABW, and the ratio by percentage of estimate within 10% of actual birthweight. Each of these error terms was average for each method of estimation in the entire study group and in the three strata of birthweights. The mean error represents the sum of the positive (overestimation) and negative (underestimation) from actual birthweight approximating zero in a method with very low or no systematic error. The difference between both the methods in the mean percentage error (i.e. the size of a systematic error) in each method was assessed by the paired *t*-test. The mean absolute percentage error is the sum of the absolute deviation (regardless of their direction) reflecting the size of the overall predictive error in terms of actual birthweight. As the absolute errors are not normally distributed, Wilcoxon signed-rank test (non-parametric) was used for testing the differences between clinical and ultrasonic estimates. The difference in proportion of estimates that are within 10% of actual birthweight was assessed by the chi-square test with p<0.05 considered as statistically significant. Each outcome measure was then assessed for overall foetal weight and for three categories of weight <2,500 g, 2,500–<4,000 g, and ≥4,000 g. The overall correlation coefficients of ultrasound based and clinically determined estimates were also compared. Data were analyzed using the SPSS (version 11.0), a windows-based statistical programme.

## RESULTS

One hundred women were recruited for the study. The mean actual birthweight of the study population was 3,254±622 (range 2,150–4,950) g. Twelve (12%) had birthweight of <2,500 g, 71% had birthweight of 2,500-<4,000 g, while 17% weighed >4,000 g.

The mean maternal age was 30.5±4.7 (range 22–41) years. The median gravidity and parity were 2 (range 1–8) and 1 (range 0–6) respectively. Thirty-five percent of gravidas were nulliparous, and 60% were multiparous, and while 5% were grandmultiparous. The mean gestational age was 38.6±1.3 (range 37–42) weeks.

**Fig. 1.**
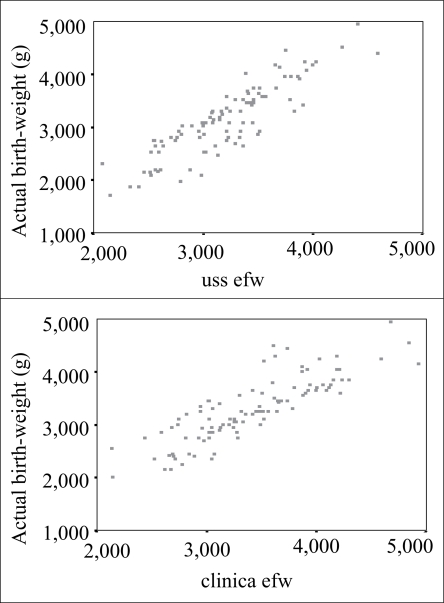
Scatter diagrams of actual birthweight by estimated foetal weight

**Fig. 2.**
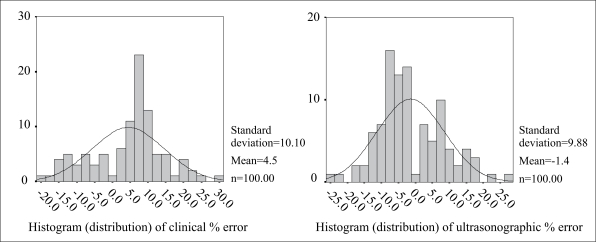
Overall distribution of percentage error terms

**Fig. 3.**
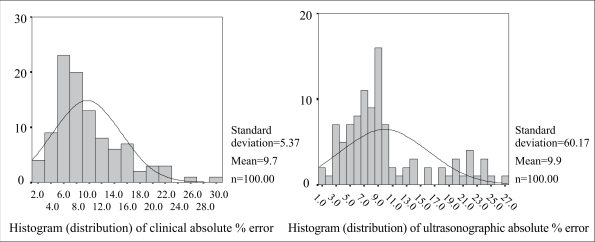
Overall distribution of absolute percentage error terms

Figure 1 shows the scatter diagram of actual birthweight by clinical and ultrasonically-estimated foetal weights. Figures 2 and 3 show the overall distribution of the error terms for the two methods. While the distribution of the percentage error is close to normal, that of the absolute percentage error is not. Table shows the accuracy and statistical differences between clinical and ultrasonically-estimated foetal weights.

clinica efw=Clinically-estimated foetal weight;
uss efw=Ultrasound scan-estimated foetal weight

**Table.** Accuracy and differences between methods of estimation

Birth-weight stratum	Clinical method	Ultrasound	p value
Overall			
Mean percentage error	4.5±10.10	-1.4±9.88	0.002*
Mean absolute percentage error	9.7±5.37	9.9±6.17	0.734†
Estimates within ABW+10%	70	68	0.760‡
Correlation coefficient	0.78	0.74	0.001**
<2,500 g			
Mean percentage error	12.5±16.2	8.9±3.1	0.030*
Mean absolute percentage error	16.1±14.6	12.6±11.7	0.063†
Estimates within ABW+10%	41.7	66.7	0.007‡
2,500-<4,000 g			
Mean percentage error	5.7±9.8	-2.2±10.1	0.000*
Mean absolute percentage error	8.9±5.9	9.2±6.3	0.729†
Estimates within ABW+10%	73.2	71.8	0.75‡
≥4,000 g			
Mean percentage error	6.5±7.2	-4.3±6.9	0.000*
Mean absolute percentage error	9.8±8.3	10.2±9.1	0.746†
Estimates within ABW+10%	70.6	64.7	0.76‡

*Pair *t-*test;

†Wilcoxon signed-rank test;

‡Chi-square test;

**Significance of Pearson's correlation coefficient

ABW=Actual birthweight

In the entire study group, the clinical method systematically overestimated actual birthweight, while the ultrasonic method underestimated it. The mean absolute percentage error was smaller for ultrasonic estimation, although the difference was not statistically significant. The number of clinical estimates that were within 10% of actual birthweight was higher than those of sonographic estimates, but the difference was not statistically significant.

In the low-birthweight (<2,500 g) group, i.e. babies with intrauterine growth restriction, both the methods systematically overestimated birthweight. All the mean errors of ultrasonic estimation were significantly smaller than those of clinical estimation, and the proportion of estimates within 10% of actual birthweight was higher for the ultrasonic method. In the middle range of birthweight (2,500-<4,000 g), the clinical method systematically overestimated birthweight. However, there was no statistically significant difference between the mean absolute percentage errors and the number of estimates within 10% of actual birthweight for the two methods.

In the high-birthweight (≥4,000 g) group, the clinical method systematically overestimated birthweight, while the ultrasonic method underestimated it. However, the difference in the means of the absolute percentage errors and the number of estimates within 10% of actual birthweight for the two methods were not statistically significant.

The correlation coefficient for the clinical and ultrasonic methods, compared to actual birthweight, were +0.78 and +0.74 respectively (Fig. 1), and results of statistical analysis showed the relationships to be statistically significant (p<0.001).

## DISCUSSION

Both foetal macrosomia and intrauterine growth restriction increase the risk of perinatal morbidity and mortality and of long-term neurologic and developmental disorders (10). Identification of intrauterine growth restriction after 37 weeks gestation is an indication for delivery to reduce the chance of foetal mortality (10,). Similarly, diagnosis of macrosomia frequently leads to delivery by means of caesarean section to reduce risk of failed vaginal delivery and shoulder dystocia (3, 4, 10).

Accurate prediction of foetal weight has been of great interest in obstetrics. As foetal weight cannot be measured directly, it must be estimated from foetal and maternal anatomical characteristics. Many workers have used different methods to achieve this. Of the various methods, the most-commonly used are the clinical and ultrasonographic methods. Only a few studies have compared the accuracy of foetal weight by clinical and ultrasonic measurements (1–7, 12, 24, 29, 34–46).

The accuracy of clinical estimation obtained in this study was highest in the birthweight range of 2,500–<4,000 g and lowest for the low-birthweight group (<2,500 g). This is in consonance with what several investigators have shown that the clinical method is best for estimating foetal weight in the reference birthweight range of 2,500 to <4,000 g with accuracy (mean absolute percentage error) of ±7.5–19.8% depending on gestational age and that below 2,500 g, accuracy of the clinical method deteriorates markedly with a mean absolute error of ±13.7–19%, and as in this study, only 40–49% of birthweights below 2,500 g threshold are estimated properly by the clinical method within 10% of actual birthweight (1, 7, 10, 22).

For the ultrasonographic method, our results are also consistent with what have been previously observed that the mean absolute percentage error of predicted birthweight varies from 6% to 12% of actual birthweight, and 40–75% of the estimates are within 10% of actual birthweight (10, 26, 29). The observation that, compared to actual birthweight, ultrasound overestimated low birthweight and underestimated high birthweight, has also been previously reported (1, 10).

In previous studies, no standardized method was used for clinical estimation, making it subjective, poorly defined, and non-reproducible. The sonographic method is widely used because it is objective and reproducible and involves a well-defined measurement procedure. In this study, we used a standardized method of clinical estimation that had been found previously to correlate well with birthweight, making it a unit protocol in our centre (21). The Hadlock (3) formula present on the ultrasound machine in our radiology unit was used for ultrasonic estimation since authors who compared the accuracy of conventionally-used formulae suggest that no single formula estimated birthweight more accurately to a significant degree than any other formulae, thus eliminating the bias that we used only Hadlock (3) formula (1).

The estimates were obtained independently by two different observers (i.e. attending senior registrars) in the obstetrics and radiology units in this study, precluding the possibility that one estimate may influence the other. The estimations were also done within 24 hours of delivery to increase the prediction power of each method.

Three measures of accuracy were used in our statistical analysis in the number of estimates within ±10% of actual birthweight, mean percentage error, and mean absolute percentage error. Interestingly, the mean percentage error can be misleading because it is the sum of positive and negative deviations from actual birthweight, thus artificially reducing the difference between actual birthweight and estimated birthweight. It is a measure of systematic error in each method and not variation from birthweight. By contrast, the mean absolute percentage error reflects the variability noted regardless of their direction and, as such, is a much more accurate predictor of differences from actual birthweight. Hence, for practical clinical purposes, the variation between predicted birthweight and actual birthweight is best expressed in the form of mean absolute percentage error (10).

The clinical measurement was confounded by the placental size and the liquor volume, which is not necessarily regarded as oligohydramnios or polyhydramnios. With ultrasound, there is an obvious limitation of comparing a spatial measurement with weight. Foetal mass is a function of foetal volume and density, and density of the foetus at term is not constant (2, 10).

The major finding from this prospective study is that clinical estimation of foetal weight is as accurate as the ultrasonographic method of estimation within the normal birthweight range. Although, while the clinical method overestimated foetal weight, our ultrasonic method underestimated it. However, when there is the case of intrauterine growth restriction (birthweight <2,500 g), both the methods overestimated birthweight, but the ultrasonic method was statistically more accurate with smaller mean errors and more estimates within ±10% of actual birthweight.

Despite the differences in study design, our findings are in consonance with those reported by others that the accuracy of clinical estimation of birthweight is similar if not better than that of ultrasonic estimation. The studies by Hendrix *et al.* and Raman *et al.* showed that clinical estimation was significantly more accurate than sonographic prediction (5, 6). Similar results as obtained by Sharman *et al.* and Titapant *et al.* who observed that ultrasonic estimation was more accurate only when there is low birthweight (1, 34) but in their own studies, both the methods underestimated birthweight by more than 400 g. Watson *et al.* found no significant difference between the two methods even at extremes of birthweight at term (7). Likewise, Baum *et al.* found no advantage of sonographic estimation over clinical or patients' estimation of foetal weight at term (29). Furthermore, Nahum and Stanislaw found that the use of ultrasonography was generally no more accurate than prediction that is based solely on quantitative assessment of maternal and pregnancy specific characteristics (30). Johnstone *et al.* also found clinical examination to be as predictive as ultrasound measurement in assessing foetal macrosomia in a diabetic population (36).

Chauhan *et al.*, in their comparison of accuracy of the two methods, observed no benefit in obtaining a sonographic estimate (2) because its accuracy is no better than that of the clinical method, except there is low birthweight (<2,500 g) when ultrasound yields a better prediction. They, however, concluded that an estimate of birthweight is associated with a wide range of actual birthweight, making obstetric decision based on such prediction to be likely associated with unnecessary intervention.

Our correlation coefficient for ultrasound estimation (0.74) is comparable with that of Uotila *et al.* in their comparison of ultrasonic estimation (0.77) with magnetic resonance imaging (0.95) in diabetic and normal pregnancy (28). The correlation coefficient of clinical estimation (0.78) is comparable to that of Dare *et al.* (0.74) in a similar population (21).

In sharp contrast to the above observation, Shamley *et al.* in 1994, comparing the clinical and ultrasonic methods, using Hadlock formula and non-standardized clinical method, noted that error of clinical estimate to be significantly higher than that for Hadlock (35). The difference from our results may be attributed to the standardized method that was used for clinical estimation in our study.

Clinical estimation of birthweight may be as accurate as routine ultrasonographic estimation, except in low-birthweight babies. Therefore, when the clinical method suggests weight smaller than 2,500 g, subsequent sonographic estimation is recommended to yield a better prediction and to further evaluate the foetal well-being.

Our observation implies that there is clearly a role for clinical estimation of birthweight as a diagnostic tool, suggesting that clinical estimation is sufficient to manage labour and delivery in a term pregnancy. Even in estimating weight of macrosomic foetus for making decision regarding trials of labour, there appears to be no benefit in obtaining a routine sonographic birthweight. The role for ultrasonographic estimation appears that, when clinically estimated weight suggests weight less than <2,500 g, subsequent sonographic estimation would yield a better prediction and would be further necessary to assess such foetuses for congenital malformation and to do the biophysical profile to determine the well-being of the foetus.

The above findings have important implication for developing countries like ours where there is lack of technologically-advanced ultrasound machines capable of doing sophisticated functions such as foetal weight but has an experienced clinician who could perform this function equally well.

The potential limitations of the study include: (a) the subjectivity of clinical estimation, (b) use of only one sonographic model to derive estimates of foetal weight, (c) no confirmation that the formula used (Hadlocks 3) is universally applicable.

We regard the overestimation of foetal weight by the clinical method as a positive factor since it will enhance the sensitivity of health workers at peripheral centres if properly taught to them for earlier referral of mothers with macrosomic foetuses, thus contributing to reduction of obstructed labour and its sequelae (47).

Further studies are, however, necessary to improve the accuracy of foetal weight and to determine if estimation of foetal weight prediction near delivery actually improves outcome and how applicable these methods can be to situations that alter birthweight such as premature rupture of membranes and obesity that were excluded in the present study.

## ACKNOWLEDGEMENTS

The hospital management is deeply appreciated for the research grant that was used for supporting the execution of this project.
